# Risk of Stroke, Thromboembolism and Mortality in Atrial Fibrillation Patients Across Different Stages and Types of Heart Failure: A Retrospective Cohort Study

**DOI:** 10.3390/jcm15114138

**Published:** 2026-05-27

**Authors:** Qinggele Gao, Yifei Wang, Yutong Zhao, Fei She, Changhua Lv, Lianfeng Liu, Peng Liu, Yuanwei Liu, Rong He, Fang Liu, Tingting Lv, Ping Zhang

**Affiliations:** 1 Department of Cardiology, Beijing Tsinghua Changgung Hospital, Tsinghua Medicine, Tsinghua University, Beijing 102218, Chinayifei-wang13@tsinghua.org.cn (Y.W.); zyta03639@btch.edu.cn (Y.Z.); sfa00820@btch.edu.cn (F.S.); lzha02095@btch.edu.cn (C.L.); llfa04942@btch.edu.cn (L.L.); wanguyisu@163.com (P.L.); lywa01945@btch.edu.cn (Y.L.); hra01560@btch.edu.cn (R.H.); lfa01077@btch.edu.cn (F.L.); 2 Department of Cardiology, The Affiliated Hospital of Inner Mongolia Medical University, Hohhot 010030, China

**Keywords:** atrial fibrillation, thromboembolism, ischemic stroke, heart failure

## Abstract

**Background/Objectives:** Atrial fibrillation (AF) and heart failure (HF) frequently coexist and worsen prognosis. The risk of stroke and thromboembolism across HF stages and types in AF patients remains incompletely clear. To evaluate the risks of stroke, thromboembolism, and all-cause mortality in AF patients stratified by HF stages (A, B, C) and types, including HF with preserved ejection fraction (HFpEF), HF with mildly reduced ejection fraction (HFmrEF), and HF with reduced ejection fraction (HFrEF). **Methods:** We conducted a retrospective cohort study of 2134 AF patients in Beijing Tsinghua Changgung Hospital. HF stages were classified per American College of Cardiology/American Heart Association (ACC/AHA) guidelines, and HF types per European Society of Cardiology (ESC) criteria. Outcomes were assessed using Kaplan–Meier and multivariable Cox regression analyses. **Results:** Over a median follow-up of 32.4 months, the incidence of stroke, thromboembolism, and mortality was significantly higher in Stage B and Stage C than in Stage A. Specifi-cally, Stage B showed higher rates than Stage A (stroke: 14.8% vs. 5.8%; thromboembo-lism: 19.1% vs. 8%; mortality: 4.0% vs. 1.2%). In Stage C, HFpEF had the highest incidence (stroke: 16.1%; thromboembolism: 22.3%; mortality: 10.3%). Adjusted models showed that the risks for all endpoints were significantly elevated in Stage B, HFpEF, and HFmrEF compared with Stage A. In contrast, HFrEF showed an elevated risk only for thromboembolism. **Conclusions:** Stage B HF was independently associated with higher risks of stroke, thromboembolism, and all-cause mortality in patients with AF. HFpEF showed the highest observed incidence of adverse outcomes among symptomatic HF phenotypes, suggesting that HF stage and phenotype may add prognostic value for AF risk stratification.

## 1. Introduction

Atrial fibrillation (AF) and heart failure (HF) are closely intertwined, with each condition predisposing to the other [1,2,3,4,5]. When present together, AF and HF are associated with a worse prognosis compared to either condition alone [3,6]. Recent guidelines, including the 2024 ESC guidelines for AF, have further refined risk stratification and management approaches for this complex patient population [7]. According to the American College of Cardiology/American Heart Association (ACC/AHA) classification system for HF [8], HF is categorized into four stages: Stage A (at risk of HF), Stage B (pre-HF), Stage C (patients with current or prior symptoms of HF), and Stage D (HF with symptoms that significantly interfere with daily life, often requiring recurrent hospitalizations despite guideline-directed medical therapy). Stage B represents a unique phase where structural or functional changes in the heart are evident, yet there are no apparent symptoms of HF.

Currently, the definition of HF is largely confined to the stages where clinical symptoms are manifest, namely, Stage C and Stage D. Therefore, the CHA_2_DS_2_-VASc score includes only these two stages of HF, whereas Stage B is often overlooked in clinical practice due to the absence of overt symptoms and signs of HF. However, previous research has shown that Stage B HF can increase cardiovascular events and cardiogenic mortality in middle-aged individuals [9]. It remains unknown whether Stage B HF increases the risk of stroke, thromboembolism, and all-cause mortality in patients with AF.

Furthermore, the recent introduction of the CHA_2_DS_2_-VA score has sparked debate on optimal risk stratification, particularly regarding the role of female sex as a risk factor [10,11]. These controversies highlight the limitations of relying solely on clinical scores and underscore the pressing need to identify more precise, pathophysiology-based risk markers.

Recent guidelines classify HF into three groups based on the left ventricular ejection fraction (LVEF): HF with reduced EF (HFrEF, EF ≤ 40%), HF with mildly reduced (HFmrEF, 40% < EF < 50%) and HF with preserved EF (HFpEF, EF ≥ 50%), as per the 2021 ESC Guidelines for the diagnosis and treatment of acute and chronic HF [12]. Most of our current understanding of the AF–HF relationship stems from studies focusing on HFrEF. In individuals aged 60 years or older, all forms of HF have a median prevalence rate of 11.8%, with HFpEF being more common than HFrEF (median prevalence of 4.9% and 3.3%, respectively) [13]. The incidence of AF is higher in HFpEF, ranging from 15% to 41%, and its presence is associated with increased mortality [14,15]. Growing evidence from registries and meta-analyses highlights the substantial risk of thromboembolism and mortality in AF patients with HFpEF, which may equal or even exceed that in HFrEF [16,17,18]. However, the prevalence of AF across different types of HF is inconsistent, and there remains debate regarding the risk of stroke and thromboembolism in patients with AF combined with either HFpEF or HFrEF [19,20,21]. This controversy is compounded by limited data specifically addressing the risk in the pre-heart failure stage (Stage B) within the AF population. Therefore, this study aimed to elucidate the risk for stroke, thromboembolism, and all-cause mortality in patients with AF and Stage B HF, as well as different types of HF.

## 2. Materials and Methods

### 2.1. Study Population

This study was conducted in accordance with the principles of the Declaration of Helsinki and was approved by the Ethics Committee of Beijing Tsinghua Changgung Hospital (review number: 21440-4-02). We performed a retrospective cohort study using a consecutive sampling strategy. A total of 2134 patients with AF treated at Beijing Tsinghua Changgung Hospital from January 2015 to December 2021 were included, including both outpatient and inpatient visits. Follow-up was conducted until 31 May 2022. For patients with multiple records, the earliest recorded visit was selected as the baseline visit.

Patients were included if they were aged ≥18 years and had documented AF on at least one 12-lead electrocardiogram or Holter monitoring record. Patients were excluded if they had valvular AF, defined as moderate-to-severe mitral stenosis or prior valve replacement surgery; incomplete baseline or follow-up data; AF secondary to reversible conditions, such as hyperthyroidism, sepsis, or postoperative status; a life expectancy of less than six months; or hematologic disorders. Because the number of patients with Stage D HF was very small (*n* = 4), these patients were excluded from the analysis. In addition, three patients with Stage B HF who progressed to Stage C HF before experiencing ischemic stroke, thromboembolism, or all-cause death were excluded to minimize potential misclassification when comparing outcomes between Stage B and Stage C HF.

### 2.2. Data Collection

Baseline data were retrieved from the hospital’s electronic medical record system. We collected demographic characteristics, medical history, and medication history, including sex, age, height, weight, date of birth, date of visit, comorbidities, and medication use. Laboratory data included estimated glomerular filtration rate (eGFR), N-terminal pro-B-type natriuretic peptide (NT-proBNP), and high-sensitivity cardiac troponin T (hs-cTnT). Echocardiographic parameters included left ventricular end-diastolic dimension (LVDD), left ventricular ejection fraction (LVEF), and left atrial (LA) diameter. The CHA_2_DS_2_-VASc score was used to assess the risk of stroke and thromboembolism, whereas the HAS-BLED score was used to assess bleeding risk.

### 2.3. Definition of HF Stages and HF Types

Patients were grouped into stages of HF according to AHA/ACC guidelines [8] and types of HF according to ESC guidelines [12]. Supplementary Material Table S1 lists the criteria used for determining the prevalence of different HF Stages and heart failure types.

### 2.4. Endpoints and Follow-Up

The primary endpoints were ischemic stroke, a composite thromboembolic endpoint, and all-cause mortality. The composite thromboembolic endpoint consisted of ischemic stroke, transient ischemic attack (TIA), and systemic thromboembolism. Ischemic stroke was defined as a new ischemic lesion detected by computed tomography or magnetic resonance imaging, or a persistent cerebrovascular neurological deficit lasting more than 24 h. Hemorrhagic stroke was not included in the primary stroke endpoint. TIA was defined as a sudden focal neurological deficit of presumed vascular origin lasting less than 24 h. Systemic thromboembolism was defined as documented loss of end-organ perfusion confirmed by imaging, surgery, or autopsy. Patients were followed until the occurrence of an endpoint event, death, or the last follow-up date of 31 May 2022, whichever came first. Events reported during telephone follow-up required confirmation by medical records or diagnostic evidence. All-cause mortality was verified through telephone interviews with relatives.

### 2.5. Statistical Analysis

Normality of continuous variables was assessed using the Shapiro–Wilk test. Normally distributed continuous variables were presented as mean ± standard deviation and were compared among groups using one-way analysis of variance. Non-normally distributed variables are presented as median and interquartile range and were compared using the Mann–Whitney U test for two-group comparisons or the Kruskal–Wallis test for comparisons among more than two groups. Categorical variables were presented as numbers (percentages, %) and compared using the χ^2^ test. Kaplan-Meier survival curves were generated to estimate event-free survival, and cumulative event rates were derived from these curves. Differences between groups were compared using the log-rank test. Multivariable Cox proportional hazards regression models were used to evaluate the associations of HF stage and HF phenotype with clinical outcomes. Results are presented as hazard ratios (HR) with 95% confidence intervals (95%CIs). To reduce potential confounding, the models were adjusted for clinically relevant covariates, including age, sex, hypertension, diabetes, prior stroke, vascular disease, dyslipidemia, hyperhomocysteinemia, left atrial diameter, and anticoagulant use. Missing baseline covariate data were handled using multiple imputation. Because the main comparisons were prespecified and hypothesis-driven, no formal correction for multiple comparisons was applied. Statistical analyses were performed using SPSS version 26.0 (IBM Corp., Armonk, NY, USA). A two-sided *p* value < 0.05 was considered statistically significant.

## 3. Results

### 3.1. Baseline Characteristics

**Figure 1:**
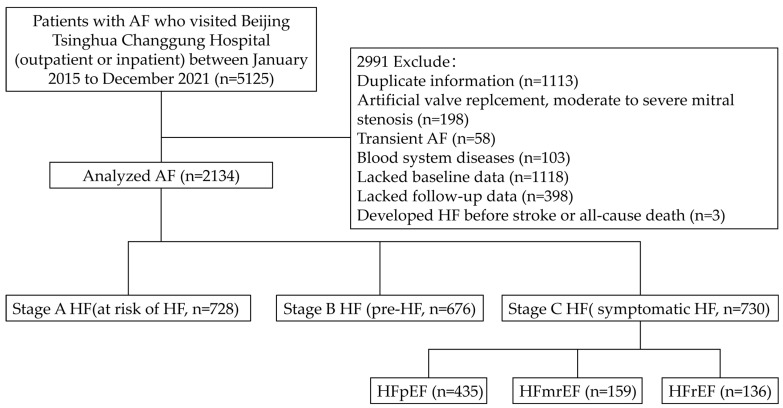
Flowchart of the Study Patients.

From January 2015 to December 2021, a total of 5125 patients with AF were treated at Beijing Tsinghua Changgung Hospital, including both outpatient and inpatient visits. Of these, 2991 patients were excluded because of duplicate records, artificial valve replacement, moderate-to-severe mitral stenosis, transient AF, hematologic disorders, missing baseline or follow-up data, or progression to heart failure before the occurrence of stroke or all-cause death. Ultimately, 2134 patients with AF were included in the final analysis. Among them, 728 patients (34.1%) were classified as Stage A HF, 676 (31.7%) as Stage B HF, and 730 (34.2%) as Stage C HF (Figure 1).

The baseline characteristics according to HF stage are presented in Supplementary Table S2. Patients with Stage C HF had the highest median age, left atrial diameter, NT-proBNP level, and CHA_2_DS_2_-VASc score, followed by those with Stage B HF and Stage A HF. Similarly, the proportions of women and patients with prior stroke were highest in the Stage C HF group, intermediate in the Stage B HF group, and lowest in the Stage A HF group. In contrast, left ventricular ejection fraction was highest in Stage A HF, followed by Stage B HF and Stage C HF. No statistically significant differences were observed among the three groups in the proportions of patients with hyperhomocysteinemia or hypertension.

### 3.2. Clinical Outcomes Across Different Stages and Types of HF in Patients with AF

**Figure 2:**
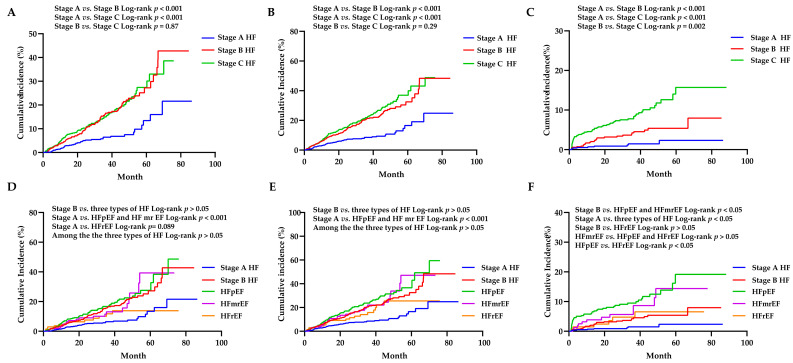
Risk Stratification by Heart Failure Stage and Type: Cumulative Incidence of Stroke, Thromboem-bolism, and All-cause Mortality. Of the 2134 AF patients analyzed, 728 had Stage A HF, 676 Stage B, and 730 Stage C. The Stage C group comprised HFpEF (n = 435), HFmrEF (n = 159), and HFrEF (n = 136). Panels (**A**–**C**) demonstrate a graded increase in the cumulative incidence of (**A**) ischemic stroke, (**B**) thromboembolism, and (**C**) all-cause mortality across advancing HF stages (**A**–**C**). Panels (**D**–**F**) further compare these risks among the pre-heart failure stage (Stage B) and the symptomatic HF phenotypes within Stage C, showing that (**D**) stroke, (**E**) thromboembolism, and (**F**) all-cause mortality were highest in patients with HFpEF. Abbreviations: HF: heart failure; HFpEF: heart failure with preserved ejection fraction; HFmrEF: heart failure with mildly reduced ejection fraction; HFrEF: heart failure with reduced ejection fraction.

During a median follow-up of 32.4 months, the incidences of ischemic stroke, thromboembolism, and all-cause mortality were 42 (5.8%), 58 (8.0%), and 9 (1.2%) in Stage A; 100 (14.8%), 129 (19.1%), and 27 (4.0%) in Stage B; and 104 (14.2%), 148 (20.3%), and 63 (8.6%) in Stage C, respectively. The corresponding incidence rates per 100 person-years were 2.3, 3.2, and 0.47 in Stage A; 5.8, 7.7, and 1.42 in Stage B; and 5.9, 8.7, and 3.22 in Stage C, respectively (Supplementary Figure S1). The Kaplan–Meier survival curves showed that the cumulative rates of stroke, thromboembolism, and all-cause mortality were higher in Stage B and Stage C compared to Stage A (Figure 2A–C). There was no significant difference in cumulative stroke or thromboembolism rates between Stage B and Stage C. However, the cumulative all-cause mortality rate was higher in the Stage C than in Stage B. Cox regression models adjusted for age, sex, hypertension, diabetes, stroke history, angiopathy, dyslipidemia, hyperhomocysteinemia, left atrial diameter, and anticoagulant use, revealed that both Stage B and Stage C had increased risks of stroke (HR 2.61; 95%CI 1.81–3.76; *p* < 0.001, HR 2.34; 95%CI 1.62–3.39; *p* < 0.001), thromboembolism(HR 2.5; 95%CI 1.82–3.45; *p* < 0.001, HR 2.49; 95%CI 1.82–3.41; *p* < 0.001), and all-cause mortality(HR 2.44; 95%CI, 1.14–5.22; *p* = 0.021, HR 5.78; 95%CI, 2.81–11.9; *p* < 0.001) compared to Stage A (Table 1, Table 2 and Table 3).

### 3.3. Baseline Characteristics of Stage C Group

The Stage C HF group was further subdivided into HFpEF, HFmrEF, and HFrEF. Of the overall cohort, 435 patients (20.4%) were classified as having HFpEF, 159 (7.4%) as having HFmrEF, and 136 (6.4%) as having HFrEF. Among the five groups, including Stage A HF, Stage B HF, HFpEF, HFmrEF, and HFrEF, patients with HFpEF had the highest median age and the highest proportions of women and dyslipidemia (*p* < 0.001, *p* = 0.002, and *p* = 0.013, respectively). In contrast, patients with HFrEF had higher proportions of diabetes, prior stroke, and vascular disease, as well as greater left ventricular end-diastolic dimension and NT-proBNP levels and lower LVEF (Supplementary Table S3).

### 3.4. Relationship Between Outcomes and Different Stages and Types of HF in Patients with AF

During a median follow-up of 32.4 months, the incidences of ischemic stroke, thromboembolism, and all-cause mortality were 42 (5.8%), 58 (8.0%), and 9 (1.2%) in Stage A HF; 100 (14.8%), 129 (19.1%), and 27 (4.0%) in Stage B HF; 70 (16.1%), 97 (22.3%), and 45 (10.3%) in HFpEF; 21 (13.2%), 31 (19.5%), and 12 (7.5%) in HFmrEF; and 13 (9.6%), 20 (14.7%), and 6 (4.4%) in HFrEF, respectively. The corresponding incidence rates per 100 person-years for ischemic stroke, thromboembolism, and all-cause mortality were 2.3, 3.2, and 0.47 in Stage A HF; 5.8, 7.7, and 1.42 in Stage B HF; 6.7, 9.7, and 3.82 in HFpEF; 5.6, 8.5, and 2.81 in HFmrEF; and 3.9, 6.3, and 1.70 in HFrEF, respectively (Supplementary Figure S2).

The Kaplan–Meier curves showed no significant differences in the cumulative incidences of ischemic stroke or thromboembolism between Stage B HF and the three symptomatic HF phenotypes, including HFpEF, HFmrEF, and HFrEF. However, the cumulative incidence of all-cause mortality was lower in Stage B HF than in HFpEF and HFmrEF. Compared with Stage A HF, the three symptomatic HF phenotypes showed higher cumulative incidences of thromboembolism and all-cause mortality, whereas cumulative ischemic stroke incidence was significantly higher in HFpEF and HFmrEF. No significant differences in cumulative ischemic stroke or thromboembolism were observed among the three HF phenotypes. However, HFpEF was associated with a higher cumulative incidence of all-cause mortality than HFrEF, while no significant differences were observed between HFpEF and HFmrEF or between HFmrEF and HFrEF (Figure 2D–F).

In multivariable Cox regression models adjusted for age, sex, hypertension, diabetes, stroke history, angiopathy, dyslipidemia, hyperhomocysteinemia, left atrial diameter, and anticoagulant use, the risk for stroke and all-cause mortality was higher in Stage B, HFpEF, and HFmrEF groups compared to Stage A. The risk of thromboembolism was significantly higher in Stage B (HR 2.51, 95% CI: 1.82–3.43, *p* < 0.001), HFpEF (HR 2.69, 95% CI: 1.92–3.76, *p* < 0.001), HFmrEF (HR 2.48, 95% CI: 1.59–3.86, *p* < 0.001), and HFrEF (HR 1.78, 95% CI: 1.04–3.05, *p* = 0.035) compared to Stage A (Table 4, Table 5 and Table 6).

## 4. Discussion

This study provides novel insights into how stroke, thromboembolism and mortality risks vary among patients with AF across different stages and phenotypes of HF. The most significant finding is that patients with Stage B HF—asymptomatic but with evident structural or functional cardiac alterations—face a substantially elevated risk of stroke, thromboembolism, and all-cause mortality compared to their Stage A counterparts. This implies that even asymptomatic structural and functional changes in the heart, characteristic of Stage B HF, predispose individuals to adverse outcomes.

In our cohort, Stage B and Stage C HF were both associated with higher risks of ischemic stroke and thromboembolism than Stage A HF, whereas no significant difference in these endpoints was observed between Stage B and Stage C. By contrast, all-cause mortality was higher in Stage C than in Stage B. These findings may suggest that subclinical structural or functional cardiac abnormalities are already associated with thromboembolic risk in patients with AF, while progression to symptomatic HF may have a stronger association with mortality. However, this interpretation should be considered hypothesis-generating, as the follow-up duration and subgroup sample sizes may have limited the ability to detect between-stage differences.

Our results demonstrate a significantly higher risk of ischemic stroke, thromboembolism, and all-cause mortality in Stage B HF relative to Stage A. This observation aligns with growing evidence underscoring the prognostic importance of subclinical cardiac injury, detectable via elevated cardiac biomarkers or subtle imaging alterations [22,23,24]. Moreover, the all-cause mortality was higher in Stage C than in Stage B HF; this suggests that progression from Stage B to Stage C HF may heighten mortality risk. However, the incidence of ischemic stroke and thromboembolism did not differ significantly between these two stages. The potential explanations for the aforementioned results are as follows. First, our study and a previous study [25] show that Stage B HF (asymptomatic structural disease) substantially increases stroke risk in AF patients. The CHA_2_DS_2_-VASc score includes heart failure as a component, whereas Stage B HF was not incorporated into this scoring system, potentially leading to insufficient anticoagulation management. Second, the duration of the follow-up period was insufficient, and the sample size was inadequate, leading to statistically insignificant differences in the results. Subsequently, we will increase the sample size and lengthen the follow-up period to conduct a prospective study aimed at further validating these findings.

Among the three types of HF (HFpEF, HFmrEF, and HFrEF), both HFpEF and HFmrEF exhibited significantly higher cumulative incidence and adjusted risks of ischemic stroke, thromboembolism, and all-cause mortality compared to Stage A HF. Notably, HFpEF showed the highest observed risk of ischemic stroke, whereas HFrEF was associated with an increased risk of thromboembolism but not ischemic stroke compared with Stage A HF. These results highlight the heterogeneity in clinical outcomes across different HF subtypes in the AF population. Our findings are consistent with recent studies such as the START registry, which identified HFpEF as a strong predictor of mortality in AF patients [17].

The elevated risks of ischemic stroke and thromboembolism in patients with HFpEF may be partly explained by left ventricular diastolic dysfunction, which has been independently associated with stroke and systemic embolism in patients with AF [15]. In contrast, left ventricular systolic function, as measured by LVEF, may be less directly related to these outcomes. This may explain why patients with HFrEF, despite reduced LVEF, did not show a significantly higher risk of ischemic stroke compared with Stage A HF [21].

Our findings are also in line with a previous study published in JACC: Clinical Electrophysiology, which reported comparable or even higher thromboembolic risk in HFpEF than in HFrEF [16]. The elevated risk in HFpEF can be further attributed to a greater burden of atrial myopathy, reflected by impaired left atrial function and reservoir strain, which is an independent predictor of stroke and mortality beyond LVEF [26,27,28]. Pathophysiological differences between HFpEF and HFrEF may further explain these differential outcomes. HFpEF is characterized by left ventricular diastolic dysfunction and reduced ventricular compliance, which increase dependence on left atrial contractility for diastolic filling. The coexistence of AF and HFpEF may aggravate these hemodynamic abnormalities and contribute to poorer outcomes.

In addition, the higher mortality observed in the HFpEF group compared to the HFrEF group may reflect differences in comorbidity profiles, anticoagulation regimens, and lower utilization of device-based therapies such as implantable cardioverter defibrillators and cardiac resynchronization therapy. In patients with HFrEF, more extensive use of guideline-directed medical therapy, including ACEIs/ARBs/ARNIs, MRAs, and SGLT2 inhibitors, as well as device-based therapies such as implantable cardioverter-defibrillators and cardiac resynchronization therapy, may modify the underlying substrate and potentially attenuate adverse outcomes [29,30]. Furthermore, left ventricular systolic function per se may be less directly linked to thrombogenesis than the diastolic dysfunction and left atrial pathology that hallmark HFpEF [31].

Although the limited response of HFpEF to conventional HF therapies is well documented [30], emerging evidence suggests that catheter ablation for AF in these HFpEF patients can significantly improve outcomes, highlighting the value of rhythm control in this phenotype [32,33,34]. Future studies should examine whether such interventions can ameliorate the high thromboembolic risk we observed.

Our findings are also consistent with those of previous studies, which have reported that a high prevalence of Stage B HF in certain population, such as midlife Black individuals (67%, median age 58 years) [9] and older adults (38% aged 65–75 years, 43% over 75 years [35]. In our cohort, 31.7% of AF patients had Stage B HF, indicating a substantial burden in this population. The higher incidence of adverse outcomes in Stage B HF compared to Stage A is consistent with prior evidence of increased cardiovascular events and mortality in Stage B HF, particularly in Black individuals [9], reinforcing the importance of identifying and managing Stage B HF, even in the absence of overt symptoms.

A systematic review and meta-analysis reported that similar stroke and systemic embolism events risks between HFrEF and HFpEF groups [16], whereas a Korean prospective cohort found that HFpEF had the highest incidence of stroke and systemic embolism among HF phenotypes [21]. These discrepancies highlight ongoing uncertainty in the field. In this context, our data support a nuanced risk-stratification approach that incorporates HF stage and phenotype, and potentially integrates advanced markers of atrial myopathy (e.g., left atrial strain) [25,36] or biomarkers (e.g., NT-proBNP) [23,37], into a more comprehensive risk assessment model.

Collectively, our data underscore that the pathophysiological continuum of HF, from subclinical alterations to symptomatic disease and across distinct phenotypes, is a critical determinant of outcomes in AF. Acknowledging this continuum mandates a refinement of our current risk prediction models. Future efforts should focus on validating the inclusion of Stage B HF and HF phenotypes into risk scores, and on exploring the value of adding biomarkers of atrial myopathy to further personalize preventive strategies. Such a multifaceted assessment holds the promise of improving survival and quality of life for the growing population of patients with AF and HF.

However, several limitations should be acknowledged. First, this was a single-center retrospective cohort study; therefore, residual confounding and selection bias could not be fully excluded despite multivariable adjustment. Second, although baseline medication history was collected and anticoagulant use was included as an adjustment variable in the multivariable models, complete longitudinal information on anticoagulant type, initial prescribed dosage, dose adjustment, temporary treatment interruption, drug switching, and long-term adherence was not available for all patients. Therefore, the potential impact of detailed anticoagulation management on ischemic stroke and thromboembolic outcomes could not be fully assessed. Third, the limited sample sizes of the HFmrEF and HFrEF subgroups, together with the median follow-up duration of 32.4 months, may have limited the statistical power for direct comparisons among HF phenotypes and for assessing long-term differences between Stage B and Stage C HF. Finally, Stage B HF was defined using available clinical, echocardiographic, and biomarker data from medical records, and our findings require validation in larger prospective multicenter cohorts.

## 5. Conclusions

In conclusion, Stage B HF was independently associated with higher risks of ischemic stroke, thromboembolism, and all-cause mortality compared with Stage A HF in patients with AF, highlighting the potential importance of early recognition and risk assessment before the onset of symptomatic HF. Among symptomatic HF phenotypes, HFpEF showed the highest observed incidence of adverse outcomes, whereas HFrEF was associated with an increased risk of thromboembolism. These findings suggest that incorporating both HF stage and HF phenotype may provide additional prognostic information for risk stratification and individualized management in patients with AF.

## Figures and Tables

**Table 1:** Multivariable Cox regression analysis of stroke risk according to heart failure stage in patients with atrial fibrillation.

	Multivariable HR (95%CI)	*p*
Stage A HF (*n* = 728)	Reference	
Stage B HF (*n* = 676)	2.61 (1.81–3.76)	<0.001 *
Stage C HF (*n* = 730)	2.34 (1.62–3.39)	<0.001 *
Female	0.96 (0.74–1.25)	0.781
Age > 60 years	2.37 (1.28–4.40)	0.006 *
Hypertension	1.21 (0.85–1.73)	0.279
Dyslipidemia	1.47 (1.09–1.97)	0.011 *
Diabetes	1.33 (1.02–1.74)	0.042 *
Vascular disease ^a^	0.77 (0.59–1.01)	0.059
Prior stroke	2.25 (1.72–2.95)	<0.001 *
HHcy	2.37 (1.76–3.19)	<0.001 *
LAD (mm)	1.02 (1.001–1.04)	0.044 *
Anticoagulant	0.75 (0.58–0.97)	0.031 *

Notes: Values are presented as median (IQR), or *n* (%) as appropriate. HHcy, hyperhomocysteinemia; LAD, left atrial dimension; ^a^ Angiographically significant coronary artery disease, previous myocardial infarction, peripheral artery disease, or aortic plaque; * *p* < 0.05.

**Table 2:** Multivariable Cox regression analysis of thromboembolism risk according to heart failure stage in patients with atrial fibrillation.

	Multivariable HR (95%CI)	*p*
Stage A HF (*n* = 728)	Reference	
Stage B HF (*n* = 676)	2.5 (1.83–3.45)	<0.001 *
Stage C HF (*n* = 730)	2.49 (1.82–3.41)	<0.001 *
Female	0.87 (0.69–1.09)	0.233
Age > 60 years	1.59 (1.02–2.39)	0.041 *
Hypertension	1.27 (0.95–1.72)	0.113
Dyslipidemia	1.31 (1.02–1.68)	0.031 *
Diabetes	1.19 (0.95–1.51)	0.132
Vascular disease ^a^	1.05 (0.84–1.34)	0.650
Prior stroke	1.78 (1.40–2.25)	<0.001 *
HHcy	2.64 (2.03–3.42)	<0.001 *
LAD (mm)	1.03 (1.01–1.04)	0.001 *
Anticoagulant	0.81 (0.65–1.01)	0.057

Notes: Values are presented as median (IQR), or *n* (%) as appropriate. HHcy, hyperhomocysteinemia; LAD, left atrial dimension; ^a^ Angiographically significant coronary artery disease, previous myocardial infarction, peripheral artery disease, or aortic plaque; * *p* < 0.05.

**Table 3:** Multivariable Cox regression analysis of all-cause mortality according to heart failure stage in patients with atrial fibrillation.

	Multivariable HR (95%CI)	*p*
Stage A HF (*n* = 728)	Reference	
Stage B HF (*n* = 676)	2.44 (1.14–5.22)	0.021 *
Stage C HF (*n* = 730)	5.78 (2.81–11.9)	<0.001 *
Female	0.89 (0.60–1.35)	0.612
Age > 75 years	2.47 (1.59–3.82)	<0.001 *
Hypertension	0.81 (0.50–1.31)	0.391
Dyslipidemia	1.13 (0.97–1.31)	0.068
Diabetes	1.07 (0.69–1.66)	0.766
Vascular disease ^a^	0.45 (0.29–0.71)	0.001 *
Prior stroke	1.34 (0.84–2.12)	0.221
HHcy	0.85 (0.46–1.58)	0.608
LAD (mm)	0.99 (0.96–1.02)	0.531
Anticoagulant	0.39 (0.25–0.61)	<0.001 *

Notes: Values are presented as median (IQR), or *n* (%) as appropriate. HHcy, hyperhomocysteinemia; LAD, left atrial dimension; ^a^ Angiographically significant coronary artery disease, previous myocardial infarction, peripheral artery disease, or aortic plaque; * *p* < 0.05.

**Table 4:** Multivariable Cox regression analysis of stroke risk according to heart failure stage and phenotype in patients with atrial fibrillation.

	Multivariable HR (95%CI)	*p*
Stage A HF (*n* = 728)	Reference	
Stage B HF (*n* = 676)	2.49 (1.68–3.69)	<0.001 *
HFpEF (*n* = 435)	2.61 (1.81–3.75)	<0.001 *
HFmrEF (*n* = 159)	2.36 (1.39–4.02)	0.002
HFrEF (*n* = 136)	1.71 (0.89–3.26)	0.110
Female	0.96 (0.74–1.25)	0.749
Age > 60 years	2.35 (1.27–4.37)	0.007 *
Hypertension	1.21 (0.85–1.72)	0.291
Dyslipidemia	1.46 (1.09–1.96)	0.011 *
Diabetes	1.33 (1.02–1.74)	0.040 *
Vascular disease ^a^	0.78 (0.59–1.02)	0.071
Prior stroke	2.26 (1.73–2.96)	<0.001 *
HHcy	2.33 (1.73–3.14)	<0.001 *
LAD (mm)	1.02 (0.94–1.10)	0.651
Anticoagulant	0.74 (0.57–0.96)	0.022 *

Notes: Values are presented as median (IQR), or *n* (%) as appropriate. HHcy, hyperhomocysteinemia; LAD, left atrial dimension; ^a^ Angiographically significant coronary artery disease, previous myocardial infarction, peripheral artery disease, or aortic plaque; * *p* < 0.05.

**Table 5:** Multivariable Cox regression analysis of thromboembolism risk according to heart failure stage and phenotype in patients with atrial fibrillation.

	Multivariable HR (95%CI)	*p*
Stage A HF (*n* = 728)	Reference	
Stage B HF (*n* = 676)	2.51 (1.82–3.43)	<0.001 *
HFpEF (*n* = 435)	2.69 (1.92–3.76)	<0.001 *
HFmrEF (*n* = 159)	2.48 (1.59–3.86)	<0.001 *
HFrEF (*n* = 136)	1.78 (1.04–3.05)	0.035 *
Female	0.86 (0.69–1.08)	0.191
Age > 60 years	1.57 (1.01–2.45)	0.047 *
Hypertension	1.28 (0.95–1.73)	0.112
Dyslipidemia	1.31 (1.02–1.67)	0.040 *
Diabetes	1.19 (0.95–1.51)	0.133
Vascular disease ^a^	1.07 (0.84–1.35)	0.592
Prior stroke	1.79 (1.41–2.27)	<0.001 *
HHcy	2.62 (2.02–3.40)	<0.001 *
LAD (mm)	1.08 (1.02–1.14)	0.008 *
Anticoagulant	0.80 (0.64–0.99)	0.040 *

Notes: Values are presented as median (IQR), or *n* (%) as appropriate. HHcy, hyperhomocysteinemia; LAD, left atrial dimension; ^a^ Angiographically significant coronary artery disease, previous myocardial infarction, peripheral artery disease, or aortic plaque; * *p* < 0.05.

**Table 6:** Multivariable Cox regression analysis of all-cause mortality according to heart failure stage and phenotype in patients with atrial fibrillation.

	Multivariable HR (95%CI)	*p*
Stage A HF (*n* = 728)	Reference	
Stage B HF (*n* = 676)	2.54 (1.15–5.23)	0.021 *
HFpEF (*n* = 435)	6.57 (3.14–13.75)	<0.001 *
HFmrEF (*n* = 159)	5.66 (2.34–13.69)	<0.001 *
HFrEF (*n* = 136)	2.82 (0.95–8.39)	0.063
Female	0.88 (0.58–1.32)	0.539
Age > 75 years	2.41 (1.55–3.74)	<0.001 *
Hypertension	0.80 (0.49–1.29)	0.360
Dyslipidemia	1.47 (0.93–2.34)	0.101
Diabetes	1.08 (0.69–1.68)	0.732
Vascular disease ^a^	1.15 (0.64–2.06)	0.633
Prior stroke	1.34 (0.85–2.13)	0.210
HHcy	0.85 (0.46–1.57)	0.589
Anticoagulant	0.39 (0.25–0.61)	0.021 *

Notes: Values are presented as median (IQR), or *n* (%) as appropriate. HHcy, hyperhomocysteinemia; ^a^ Angiographically significant coronary artery disease, previous myocardial infarction, peripheral artery disease, or aortic plaque; * *p* < 0.05.

## Data Availability

De-identified data is available from the corresponding authors upon reasonable request and with permission from the Ethics Committee of Beijing Tsinghua Changgung Hospital.

## Supplementary Materials

The following supporting information can be downloaded at: https://www.mdpi.com/article/10.3390/jcm15114138/s1, Figure S1: Incidence of stroke, thromboembolism and all-cause mortality in different stages of heart failure/100 person-years. Figure S2: Incidence of stroke in stage A HF, stage B HF and different types of heart failure. Table S1: Criteria used for determining Heart Failure stage and heart failure type. Table S2: Baseline Characteristics of patients with atrial fibrillation combined with different stages of heart failure. Table S3: Baseline Characteristics of patients with atrial fibrillation combined with stage A, stage B and different types of heart failure.

## Abbreviations

The following abbreviations are used in this manuscript:

ACC/AHA	American College of Cardiology/American Heart Association
AF	Atrial fibrillation
eGFR	Estimated glomerular filtration rate
ESC	European Society of Cardiology
HF	Heart failure
HFmrEF	Heart failure with mildly reduced ejection fraction
HFpEF	Heart failure with preserved ejection fraction
HFrEF	Heart failure with reduced ejection fraction
hs-cTnT	High-sensitivity cardiac troponin T
LA	Left atrium
LVEF	Left ventricular ejection fraction
LVDD	Left ventricular end-diastolic dimension
NT-proBNP	N-terminal pro-B-type natriuretic peptide
