# Supplementary material for: Risk of Stroke, Thromboembolism and Mortality in Atrial Fibrillation Patients Across Different Stages and Types of Heart Failure: A Retrospective Cohort Study
Source: J Clin Med. 2026 May 27;15(11):4138. doi: 10.3390/jcm15114138 (PMC13257787; doi:10.3390/jcm15114138)
Supplement: Supplementary file 1 [file jcm-15-04138-s001.zip › jcm-4298904-supplementary.pdf]

Supplementary Material

Figure S1. Incidence of stroke, thromboembolism and all-cause mortality in different stages of heart failure/100 person-years. A. The incidence rates of stroke in the three groups were 2.3/100 person years in the Stage A, 5.8/100 person years in the Stage B, and 5.9/100 person years in the Stage C, respectively. **B.** The incidence rates of thromboembolism in the three groups were 3.2/100 person years in the Stage A, 7.7/100 person years in the stage B, and 8.7/100 person years in the stage C. **C.** The all-cause mortality rates in the three groups were 0.47/100 person years in the Stage A, 1.42/100 person years in the Stage B, and 3.22/100 person years in the stage C, respectively.

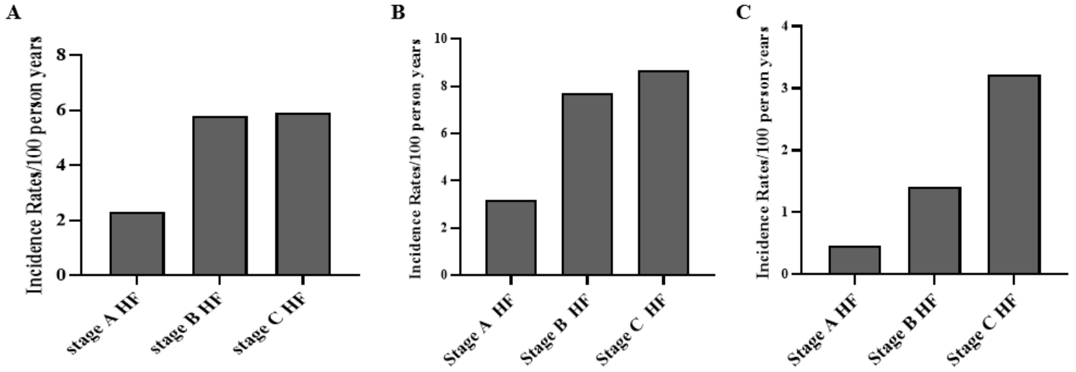

Figure S2 . Incidence of stroke in stageA HF, stageB HF and different types of heart failure. **A.** Incidence of stroke in stageA HF, stageB HF and different types of heart failure/100 person-years. The incidence rates of stroke in the five groups were 2.3, 5.8, 6.7, 5.6, 3.9/100 person year in stage A, stage B, HFpEF, HFmrEF, and HFrEF group, respectively. **B.** Incidence of thromboembolism in stageA HF, stageA HF and different types of heart failure/100 person-years. The incidence rates of thromboembolism in the five groups were 3.2, 7.8, 9.7, 8.5 and 6.3/100 person years in stage A, stage B, HFpEF, HFmrEF, and HFrEF group, respectively. **C.** Incidence of all-cause mortality in stageA HF, stageA HF and different types of heart failure/100 person-years; The incidence rates of all-cause death in the five groups were 0.47, 1.42, 3.82, 2.81, and 1.7/100 person years in stage A, stage B, HFpEF, HFmrEF, and HFrEF group, respectively.

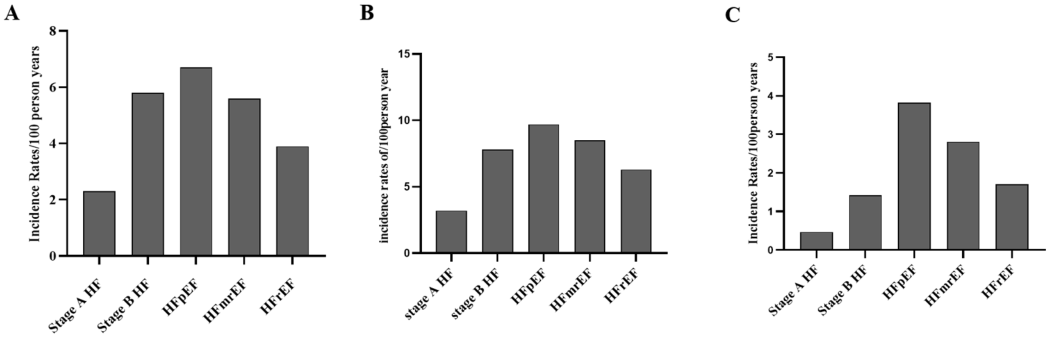

Table S1. criteria used for determining Heart Failure stage and heart failure types

HF stage	Criteria for Definition
Stage A	Presence of at least 1 of the following HF risk factors but without current or previous symptoms of HF and without structural/functional heart disease or abnormal biomarkers:<br>• Hypertension (SBP/DBP ≥140/90 mm Hg)<br>• Diabetes mellitus (fasting glucose ≥126 mg/dL or use of diabetes mellitus medication)<br>• Obesity (BMI ≥30 kg/m2)

	• Coronary artery disease (excluding myocardial infarction) but no cardiac structural/functional abnormality on imaging studies<br>•exposure to cardiotoxic agents,genetic variant for cardiomyopathy,or family history of cardiomyopathy
Stage B	Patients without current or previous symptoms of HF but evidence of 1 of the following:<br>• Prior clinical myocardial infarction<br>• Echocardiographic evidence of asymptomatic left ventricular systolic dysfunction: left ventricular ejection fraction of <0.50<br>• Left ventricular hypertrophy (LVH)=LVPW≥12mm<br>•NT-proBNP>660pg/ml<br>• Left ventricular enlargement: female>50mm,male>55mm
Stage C	patients with current or previous symptoms of HF:<br>**HFpEF:** LVEF>50% and evidence of 1 of the following:<br>•Left Atrial enlargement :female>38mm,male>40mm;<br>•Left ventricular enlargement: female>50mm,male>55mm;<br>•Left ventricular hypertrophy (LVH)=LVPW≥12mm;<br>•NT-proBNP>660pg/ml;<br>**HFmrEF:** LVEF40%-49% and NT-proBNP>660pg/ml;<br>**HFrEF:** LVEF<40%;

Table S2. Baseline Characteristics of patients with atrial fibrillation combined with different stages of heart failure

	StageA (n=728)	StageB (n=676)	StageC (n=730)	*P*
Female	272 (37.4)	275 (40.7)	318 (43.6)	0.034
Age(years)	69 (63-77)	73 (65-80)	75 (67-83)	<0.001*
age＞60years	600 (82.4)	588 (87)	648 (88.8)	0.002*

Hypertension	514 (70.6)	487 (72.0)	536 (73.4)	0.480
Diabetes	205 (28.2)	164 (24.3)	279 (38.2)	<0.001*
Dyslipidemia	359 (49.3)	309 (45.5)	395 (54.1)	0.005*
HHcy	67 (9.2)	69 (10.2)	85 (11.6)	0.291
Prior stroke	104 (14.3)	124 (18.3)	176 (24.1)	<0.001*
vascular disease[a]	278 (38.2)	228 (33.7)	408 (55.9)	<0.001*
LVEF (%)	64 (61-65)	62 (60-65)	55 (43-60)	<0.001*
LVEDD (mm)	48 (45-50）	48 (45-52 ）	50 (46-55）	<0.001*
LAD (mm)	38 (36-42)	41 (38-46)	44 (40-50)	<0.001*
NT-proBNP(pg/ml)	219 (109-399)	1393 (847-2325)	2336 (1082-5770)	<0.001*
Anticoagulant	403 (55.4)	326 (48.2)	395 (54.1)	<0.001*
HAS-BLED	1 (1-2)	1 (1-2)	2 (1-3)	<0.001*
CHA2DS2-VASC Score	3 (2-4)	3 (2-4)	5 (4-6)	<0.001*
0 Score	40 (5.5)	27 (4)	0 (0)	
1 Score	89 (12.2)	75 (11)	25 (3.4)	
≥2 Score	599 (82.3)	574 (84.9)	705 (96.6)	

Notes: Values are presented as median (IQR), or n (%) as appropriate. HHcy, hyperhomocysteinemia; LVEF, left ventricular ejection fraction; LVDD, left ventricular end diastolic dimension; LAD, left atrial dimension; hsTnT, Troponin T high-sensitivity; NT -proBNP, N-terminal pro-B-type natriuretic peptide; eGFR, estimated glomerular filtration rate; SBP, systolic blood pressure; DBP*, diastolic* blood pressure; a, Angiographically significant coronary artery disease, previous myocardial infarction, peripheral artery disease, or aortic plaque; *$P<0.05$.

Table S3. Baseline Characteristics of patients with atrial fibrillation combined with stageA, stageB and different types of heart failure

	Stage A (n=728)	Stage B(n=676)	HFpEF(n=435)	HFmrEF(n=159)	HFrEF (n=136)	*P*
female	272 (37.4)	275 (40.7)	211 (48.5)	60 (37.7)	47 (34.6)	0.002*
Age(years)	69 (63-77)	73 (65-80)	77 (70-84)	73 (65-79)	72 (64-79)	<0.001*
age＞60years	600 (82.4)	588 (87)	397 (91.3)	138 (86.8)	113 (83.1)	0.001*
Hypertension	514 (70.6)	487 (72.0)	332 (76.3)	111 (69.8)	93 (68.4)	0.189
Diabetes	205 (28.2)	164 (24.3)	166 (38.2)	60 (37.7)	53 (39.0)	<0.001*
Dyslipidemia	359 (49.3)	307 (45.4)	245 (56.3)	80 (50.3)	70 (51.5)	0.013*
HHcy	67 (9.2)	69 (10.2)	50 (11.5)	23 (14.5)	12 (8.8)	0.302
Prior stroke	104 (14.3)	124 (18.3)	106 (24.4)	36 (22.6)	34 (25.0)	<0.001*
vascular disease [a]	278 (38.2)	228 (33.7)	226 (52)	93 (58.5)	89 (65.4)	<0.001*
LVEF (%)	64 (61-65)	62 (60-65)	60 (58-63)	45 (42-47)	34 (28-37)	<0.001*
LVEDD (mm)	48 (45-50)	48 (45-52)	48 (45-51)	53 (49-56)	59 (54-64)	<0.001*
LAD (mm)	38 (36-42)	41 (38-46)	44 (40-49)	45 (42-49)	45 (41-50)	<0.001*

Continue Table S3. Baseline Characteristics of patients with atrial fibrillation combined with stageA, stageB and different types of heart failure

	StageA (n=728)	StageB（n=676）	HFpEF (n=435)	HFmrEF (n=159)	HFrEF (n=136)	*P*
NT-proBNP (pg/ml)	219 (109-399)	1393 (847-2325)	2542 (1157-5799)	1784 (933-4013)	2703 (1013 - [illegible]	<0.001*
Anticoagulant	403 (50.4)	326 (48.2)	232 (53.3)	87 (54.7)	76 (55.9)	0.079
CHA2DS2-VASC Score	3 (2-4)	3 (2-4)	5 (4-6)	5 (3-6)	5 (3-6)	<0.001*
0 Score	40 (5.5)	27 (4)	0 (0.0)	0 (0.0)	0 (0.0)	<0.001*
1 Score	89 (12.2)	75 (11)	8 (1.8)	9 (5.7)	8 (5.9)	
≥2 Score	599 (82.8)	574 (84.9)	427 (98.2)	150 (94.3)	128 (94.1)	
HAS-BLED	1 (1-2)	1 (1-2)	2 (1-3)	2 (1-3)	2 (1-3)	<0.001*

Notes: Values are presented as median (IQR),or n (%) as appropriate. HHcy, hyperhomocysteinemia;LVEF, left ventricular ejection fraction; LVDD, Left ventricular end diastolic dimension; LAD, left atrial dimension; hsTnT, Troponin T high-sensitivity; NT -proBNP, N-terminal pro-B-type natriuretic peptide; eGFR, estimated glomerular filtration rate; SBP, systolic blood pressure; DBP, *diastolic* blood pressure; a, Angiographically significant coronary artery disease, previous myocardial infarction, peripheral artery disease, or aortic plaque; $^*P$<0.05.
